# Lipidation states orchestrate CLICK-III/CaMKIγ's stepwise association with Golgi and rafts-enriched membranes and specify its functional coupling to STEF-Rac1-dependent neurite extension

**DOI:** 10.3389/fncel.2023.1204302

**Published:** 2023-08-03

**Authors:** Natsumi Ageta-Ishihara, Sayaka Takemoto-Kimura, Yayoi Kondo, Michiko Okamura, Haruhiko Bito

**Affiliations:** ^1^Department of Biomolecular Science, Faculty of Science, Toho University, Funabashi, Japan; ^2^PRESTO, Japan Science and Technology Agency, Kawaguchi, Japan; ^3^Department of Neurochemistry, Graduate School of Medicine, The University of Tokyo, Bunkyo, Japan; ^4^Department of Neuroscience I, Research Institute of Environmental Medicine, Nagoya University, Nagoya, Japan

**Keywords:** palmitoylation, CaMKIγ, Golgi apparatus, lipid rafts, neuritogenesis

## Abstract

CLICK-III/CaMKIγ is a lipid-anchored neuronal isoform of multifunctional Ca^2+^/calmodulin-dependent protein kinases, which mediates BDNF-dependent dendritogenesis in cultured cortical neurons. We found that two distinct lipidation states of CaMKIγ, namely, prenylation and palmitoylation, controlled its association with detergent-resistant microdomains in the dendrites and were essential for its dendritogenic activity. However, the impact of each lipid modification on membrane targeting/trafficking and how it specifies functional coupling leading to polarized changes in neuronal morphology are not clear. Here, we show that prenylation induces membrane anchoring of CaMKIγ, permitting access to the Golgi apparatus, and a subsequent palmitoylation facilitates association with cholesterol-enriched lipid microdomains or lipid rafts, in particular at the Golgi. To specifically test the role of palmitoylated CaMKγ in neurite extension, we identified and took advantage of a cell system, PC12, which, unlike neurons, conveniently lacked CaMKIγ and was deficient in the activity-dependent release of a neuritogenic growth factor while possessing the ability to activate polarized rafts signaling for morphogenesis. This system allowed us to rigorously demonstrate that an activity-dependent, lipid rafts-restricted Rac activation leading to neuritogenesis could be functionally rescued by dually lipidated CaMKIγ expression, revealing that not only prenylation but also palmitoylation is essential for CaMKIγ to activate a compartmentalized STEF-Rac1 pathway. These results shed light on the significance of recruiting prenylated and palmitoylated CaMKIγ into the coalescing signalosomes at lipid rafts together with Rac1 and its specific GEF and STEF and forming a compartmentalized Ca^2+^ signaling pathway that underlies activity-dependent neuritogenesis and morphogenesis during axodendritic polarization critical for brain development and circuitogenesis.

## Introduction

Multifunctional Ca^2+^/calmodulin-dependent protein kinases, CaMKI, II, and IV, are involved in a wide range of neuronal changes in response to intracellular Ca^2+^ elevation, which is critical for the proper development and function of the nervous system (Hook and Means, 2001; Soderling and Stull, 2001; Hudmon and Schulman, 2002). Several studies have demonstrated that the CaMKK-CaMKI pathway regulates neuronal morphogenesis in cultured cortical and hippocampal neurons as well as in model cell lines, drawing attention to it as a potential key player that mediates activity-dependent neuronal morphogenesis (Takemoto-Kimura et al., 2007; Saneyoshi et al., 2008).

CLICK-III/CaMKIγ is a neuronal isoform of the CaMKI family (Takemoto-Kimura et al., 2003), which has been shown to mediate BDNF-dependent dendritogenesis (Takemoto-Kimura et al., 2007) and axon formation (Davare et al., 2009) in cultured neurons. One unique feature of CLICK-III/CaMKIγ is membrane association via two distinct lipid modifications—prenylation and palmitoylation. Prenylation occurs on its C-terminal CaaX motif, followed by palmitoylation on adjacent cysteine residues, presumably via neuronal DHHC palmitoyl transferases such as DHHC3/GODZ (Takemoto-Kimura et al., 2003, 2007). These post-translational lipid modifications of CLICK-III/CaMKIγ play an essential role in directing its membrane association and its further targeting into the detergent-resistant microdomain in the dendrites. Moreover, these lipid modifications are necessary for CaMKIγ to induce dendritic outgrowth, a critical morphogenetic event for determining axodendritic polarity in cortical neurons (Takemoto-Kimura et al., 2007). However, how each lipid modification (prenylation and palmitoylation) underlies specific steps in CaMKIγ's subcellular distribution and how its enhanced assembly to lipid raft membranes determines functional coupling to downstream cytoskeletal signaling pathways are not clear.

Lipid-anchored proteins are often modified with several distinct lipidation steps such as prenylation, myristoylation, and palmitoylation (Fivaz and Meyer, 2003). While prenylation and myristoylation are mediated by soluble enzymes co-translationally, palmitoylation is a post-translational lipid modification that takes place mainly in the Golgi apparatus. Palmitoylation is largely driven by DHHC proteins, a family of palmitoyl acyl transferases (PATs) (Fukata and Fukata, 2010; Rocks et al., 2010; Greaves and Chamberlain, 2011). Then, palmitoylated proteins are shown to be trafficked to the peripheral plasma membranes and localize to lipid raft membranes, while depalmitoylation seems to occur globally (Rocks et al., 2010). Thus, unlike other lipid modifications, palmitoylation is unstable and reversible, and the dynamic cycles of palmitoylation-depalmitoylation act as a signaling switch that recruit proteins transiently to the appropriate signaling nanoclusters (Linder and Deschenes, 2007; Salaun et al., 2010). Furthermore, heightened neuronal activity regulates palmitoylation-depalmitoylation cycles (Kang et al., 2008), thus determining the polarized trafficking and function of many proteins (El-Husseini Ael et al., 2002; Huang and El-Husseini, 2005; Kang et al., 2008; Hayashi et al., 2009) in response to neuronal activity.

In this report, we demonstrated that palmitoylation of CaMKIγ via GODZ, a non-raft Golgi protein, is sufficient and essential for CaMKIγ's biochemical fractionation into lipid raft membrane microdomains. Interestingly, prenylation is sufficient for CaMKIγ's membrane anchoring, especially at the Golgi apparatus, but a lack of palmitoylation does not affect CaMKIγ's localization to the Golgi. Consistent with its delivery to lipid raft membranes, the coalescence of multiple CaMKIγ molecules was promoted by palmitoylation. However, the lack of any lipid modification had less effect on its regulated Ca^2+^/CaM-dependent kinase activity. To demonstrate the critical significance of recruiting CaMKIγ into rafts in order to elicit a robust morphogenetic response such as neuritogenesis, we took advantage of PC12, a cell line lacking CaMKIγ but possessing multiple signal-dependent neuritogenetic pathways that partly relied upon lipid rafts. Functional rescue experiments revealed that palmitoylation is essential for the functional coupling of CaMKIγ with the downstream STEF-Rac1 pathway to induce activity-induced neurite extension. These results suggest that palmitoylated CaMKIγ is preferentially recruited into the coalescing signalosomes at the lipid rafts and specifically couples with STEF to trigger a polarized Rac1 response, critical for Ca^2+^-dependent neuronal morphogenesis.

## Materials and methods

### Cloning and plasmid constructions

Mouse CaMKIγ cDNA was subcloned into pEGFPC1 (BD Clontech) and pcDNA3 (Invitrogen), and mutants were generated by stepwise substitution of each codon by site-directed mutagenesis. GODZ cDNA (a kind gift from Dr. Masayoshi Mishina, University of Tokyo, Japan) (Uemura et al., 2002) was subcloned into pcDNA3-HA (Takemoto-Kimura et al., 2003). pEGFP-V12Rac1 was generated by site-directed mutagenesis from pEGFP-N17Rac1 (a kind gift from Dr. Shuh Narumiya, Kyoto University, Japan), and pcDNA3-Flag-PHnTSS STEF (N-terminal PH domain and Tiam-STEF-SIF homologous domain as a dominant negative for STEF/Tiam2) was generated as described previously (Matsuo et al., 2002).

### Antibodies

We used commercial rat and mouse antibodies: anti-GFP (Nacalai), anti-HA (Roche Diagnostics, Cell Signaling), anti-Flotillin-1, anti-Caveolin-1, anti-Rac1, anti-GM130 (BD), horseradish peroxidase-conjugated IgG (Amersham Biosciences), and Alexa 488-, Alexa 555-, and Alexa 594-conjugated IgG (Molecular Probes).

### Cell culture and drug treatment

COS-7 cells were maintained in Dulbecco's modified Eagle medium (DMEM), containing 10% heat-inactivated fetal calf serum (FCS). PC12 cells were grown in DMEM containing 10% heat-inactivated horse serum (HS) and 5% heat-inactivated FCS using a PRIMARIA culture dish (BD Falcon).

**Figure 1:**
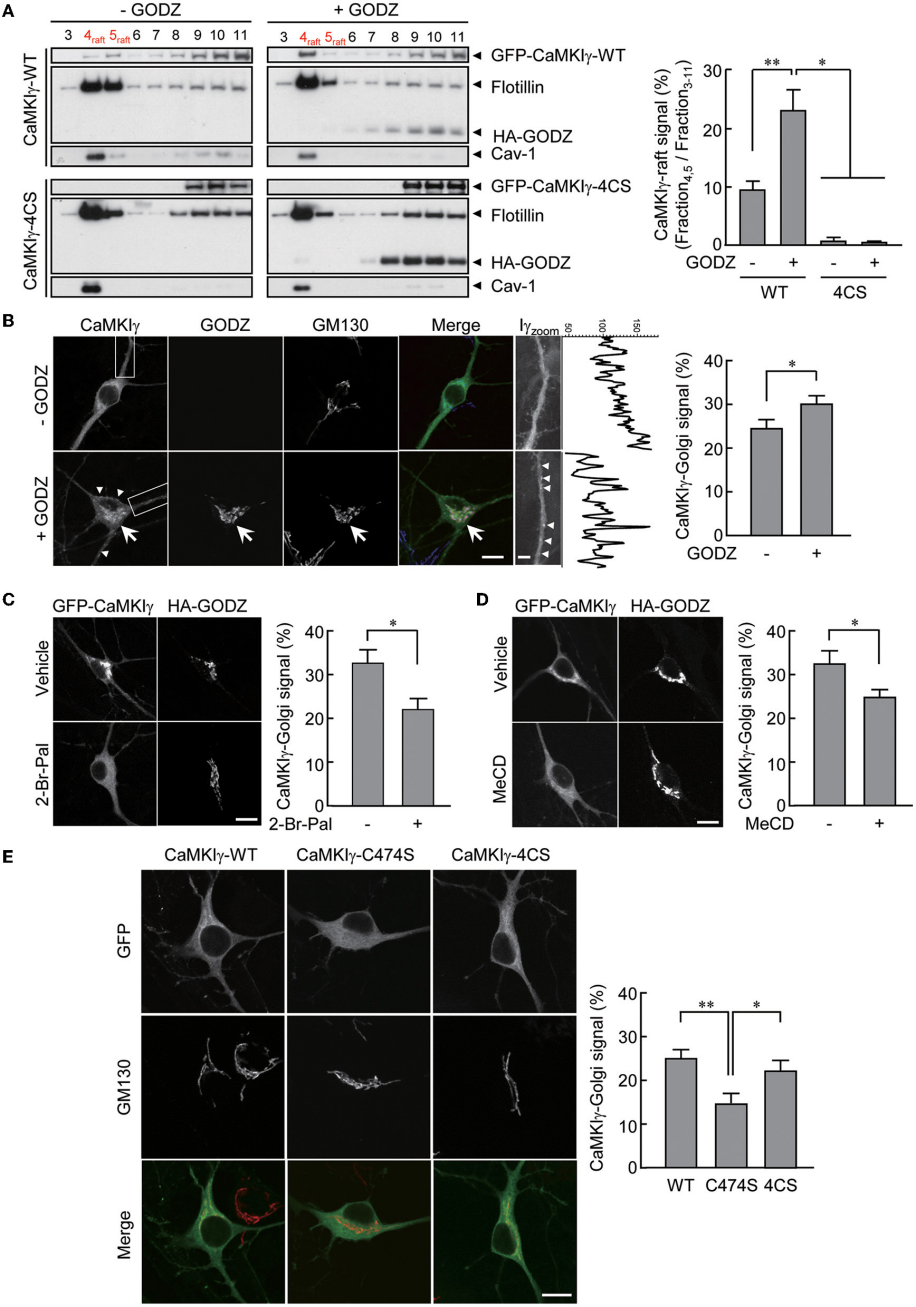
An essential role of palmitoylation in lipid raft association. **(A)** Recovery of wild-type GFP-CaMKIγ-WT in the lipid raft fraction (upper left, lane 4–5) was augmented by an overexpression of HA-GODZ (upper right, lane 4–5). In contrast, palmitoylation-deficient GFP-CaMKIγ-4CS was not detected in the lipid raft fraction irrespective of GODZ overexpression (lower panels, lane 4–5). Note that GODZ was not recovered in the lipid rafts. The lipid raft fractions were confirmed by raft proteins, caveolin-1 (Cav-1) and flotillin. *n* = 3, 3, 3, 3. **(B)** HA-GODZ expression induced strong punctate accumulation of GFP-CaMKIγ at the Golgi complex [**(B)**, arrow, stained with GM130] as well as at the plasma membranes in the dendrites and the soma [**(B)**, arrowheads] in cultured hippocampal neurons. Without HA-GODZ expression, GFP-CaMKIγ was localized predominantly to the Golgi membranes, while some proportion remained distributed relatively diffusely. Iγ_zoom_ was the magnified image of CaMKIγ in the dendrites enclosed by dotted squares. Fluorescence line scan profiles of CaMKIγ along the dendrite revealed more prominent peak amplitudes in the presence of a significant cluster of CaMKIγ (+GODZ). *n* = 8, 9. **(C, D)** Inhibition of palmitoylation by 2-bromopalmitate (2-Br-Pal) or depletion of cholesterol by methyl-beta-cyclodextrin (MeCD) abolished GODZ-induced punctate accumulation of CaMKIγ. In contrast, GODZ distribution remained restricted to the Golgi apparatus irrespective of the presence of 2-bromopalmitate or methyl-beta-cyclodextrin (MeCD). Single confocal images are shown. **(C)** *n* = 4, 3. **(D)** *n* = 4, 5. **(E)** GFP-fused prenylation and palmitoylation deficient mutants (C474S) are distributed diffusely throughout the cytoplasm, while palmitoylation deficient mutants (4CS) show similar localization with the WT, marked by colocalization with the Golgi marker GM130. *n* = 7, 6, 7. Scale bars: 10 μm **(B–E)**, 1 μm **(B)**. *p < 0.05; **p < 0.01.

Mouse hippocampal neurons were prepared from P0 ICR mice, as described previously (Bito et al., 1996). Dissociated cells were plated onto 12-mm Matrigel-coated coverslips, transfected with each expression vector using Lipofectamine 2000 (Invitrogen) at 7 days *in vitro*, and fixed 2–3 days later for immunostaining. Neurons were treated with either 60 μM 2-bromopalmitate (Sigma) overnight or 10 mM methyl-β-cyclodextrin (Sigma) for 20 min before fixation (Figures 1C, D).

### Immunocytochemistry

Cells were fixed in 4% paraformaldehyde/4% sucrose/phosphate-buffered saline (PBS)(–) for 20 min and washed with 0.1 M glycine/PBS(–). Immunostaining was carried out as described (Bito et al., 1996; Takemoto-Kimura et al., 2003, 2007; Ageta-Ishihara et al., 2009). Fluorescence imaging was performed using scanning laser confocal microscopes (LSM 510 and LSM900 with Airyscan 2, Zeiss) with 63× and 40× objective lenses (Plan-Apochromat 63× /NA 1.4, oil; Plan-Neofluar 40× /NA 1.3, oil, Zeiss) and wide-field microscopes (BX-51, Olympus) with 20× objective lenses (UplanFI 20× /NA 0.5, air, Olympus) and a color CCD camera (DP-70, Olympus). Projected images of confocal sections are shown, but occasionally, for clear separation of membrane- and/or Golgi-fluorescent signals, single confocal sections are shown (Figures 1B–E). Fluorescence intensity was measured using Fiji (ImageJ). The localization of CaMKIγ to the Golgi was calculated as follows: (%) = (the summation of the GFP-CaMKIγ signal in GM130 (a marker of the Golgi-positive area)/(the summation of the GFP-CaMKIγ signal in the soma area) × 100).

### Co-immunoprecipitation, lipid raft fractionation, and western blot analysis

For co-immunoprecipitation experiments, COS-7 cells were transfected with wild-type and mutant GFP-CaMKIγ vectors and wild-type HA-CaMKIγ using the Fugene6 reagent (Roche Diagnostics). Cell lysates were prepared 36 h after transfection, and immunoprecipitation was performed as follows. Cells were washed twice in PBS(–), lysed with lysis buffer [50 mM Tris-HCl pH 7.5, 100 mM NaCl, 2 mM MgCl_2_, 10% Glycerol, 1% Triton X-100, and protease inhibitors (Roche Diagnostics)], and mixed with protein A Sepharose (Amersham Biosciences) and GFP antibody (Molecular Probes). Each sample was mixed with an equal amount of 4× sample buffer (0.2 M Tris-HCl pH 6.8, 8% SDS, 40% glycerol, 40% 2-mercaptoethanol, and 0.02% bromophenol blue) and boiled for 3 min and was subjected to Western blot analysis.

Detergent-insoluble membrane fractions were obtained according to a previously described method (Morishima-Kawashima and Ihara, 1998; Takemoto-Kimura et al., 2007). Briefly, COS-7 cells cultured in three 10-cm dishes were transfected with the expression vectors, harvested 2 days later, and homogenized in 2.5 ml of MES-buffered saline (MBS; pH 6.5), containing 1% Triton X-100 and protease inhibitors (Roche Diagnostics). These obtained lysates were adjusted to a final 40% sucrose concentration in MBS, and 4 ml of each lysate was placed at the bottom of an ultracentrifuge tube and overlaid with a discontinuous sucrose gradient consisting of 4 ml of 5%/4 ml of 35% in MBS. Ultracentrifugation was carried out at 39,000 rpm for 20 h at 4°C in an SW 41 rotor (Beckman). A total of 12 fractions (1 ml each) and an extract of the resultant pellet were collected along with 15 μl of each fraction.

Western blot analysis was carried out as described (Takemoto-Kimura et al., 2007; Ageta-Ishihara et al., 2009). Films were digitally scanned, and rectangular windows of an identical area were defined around each band of interest. After background subtraction, the average pixel intensity of each region of interest was calculated using ImageJ 1.36 (NIH) and Fiji (ImageJ).

### Immunoprecipitate kinase assay

COS-7 cells were transfected with GFP-CaMKIγ or GFP-CaMKIγ-C474S and Myc-CaMKKactive or an empty vector using Lipofectamine 2000. After transfection for 48 h, an immunoprecipitate kinase assay was performed using MBP as a substrate (Takemoto-Kimura et al., 2003). CaMKIγ immunopurification was evaluated by silver staining using a PlusOne Silver Staining Kit (Amersham). CaMKK expression was functionally confirmed by the similar intensity of the phosphorylated CaMKIγ band under extended exposure.

### RNA extraction and RT-PCR

Total RNAs were extracted from the P12 rat brain and PC12 cells using Trizol (Life Technologies). After DNase treatment, cDNAs were synthesized from 2 μg of total RNA using the Omniscript RT Kit (Qiagen). PCR reactions were performed using the following primers:

- CaMKKα forward: 5′-CTC AGG GAG GGC CAG CCA AAC AGC T-3′
- CaMKKα reverse: 5′-CCT GCC GTA CTG GAC AGC TGA GCA T-3′
- CaMKKβ forward: 5′-GTT CCC ACC CTC AAG CCA CTG TCT G-3′
- CaMKKβ reverse: 5′-CTG GAA ACT CCA GGG CCT GAC TC-3′
- CaMKIα forward: 5′-TCC TGG CCC AGA AGC CCT ACA GCA-3′
- CaMKIα reverse: 5′-CAT GTG CCG AAC CAC AGC GGT AGC A-3′
- CaMKIβ forward: 5′-TGA TGC TGG CCC AGG AAA GGG GCT-3′
- CaMKIβ reverse: 5′-TCC CGG TGC ACG ATG CCC AGG CTA T-3′
- CaMKIγ forward: 5′-CCT GCT GTA CCT CAC CCC TGA GGA G-3′
- CaMKIγ reverse: 5′-AGA TGG GTA GAT GTC CCG GTG CAG G-3′
- CaMKIδ forward: 5′-ATC GCT GGT GAC ACA GCC CTC AGC A-3′
- CaMKIδ reverse: 5′-TTG TCA CAG TGG TGG GCC TGG GTC T-3′
- CaMKIV forward: 5′-CAA CGC CAG CCC CTG ATG CAC CAC T-3′
- CaMKIV reverse: 5′-TGA CCC ACG GGT GTT GGA GGG CTT-3′
- GAPDH forward: 5′-TGA ACG GGA AGC TCA CTG G-3′
- GAPDH reverse: 5′-TCC ACC ACC CTG TTG CTG TA-3′

### Measurement of neuritogenic activity and Rac1 pulldown assays using PC12 cells

PC12 cells, previously used for studies of small GTPases (kind gift from Dr. Shuh Narumiya, Kyoto University, Japan), were grown onto poly-Lysine-coated glass coverslips or culture dishes (BD Biosciences).

For a neuritogenic activity assay, wild-type or mutant HA-CaMKIγ vectors were co-transfected with pEGFPC1 as a morphological marker using Optifect reagent (Invitrogen). Alternatively, in experiments employing co-transfection of another signaling molecule, such as GFP-N17Rac1 or Flag-PHnTSS STEF, cell contours were reliably identified using DIC images. Transfected PC12 cells were treated with a high K^+^ medium containing 55 mM KCl at either 4 h or 24 h after transfection, kept for another 24 h, fixed, and immunostained for morphological analyses. We defined neuritogenic activity by counting the percentages of cells bearing at least one neurite with a length that exceeded the larger diameter of the cell body (neurite-positive cells) within many randomly chosen fields of view. We excluded from our analyses all cells in which either one of the co-transfected molecules was not detected. An observer blind to the transfection type counted the number of neurite-positive cells. In each experiment, an average percentage of the neurite-positive cells from two coverslips was obtained for each construct (*N* > 500 transfected cells per coverslip). The shown results are means ± SEM from three to five independent experiments.

For Rac1 pulldown assays, PC12 cells were transfected with wild-type or mutant HA-CaMKIγ vectors using the Lipofectamine 2000. After 24 h, the cells were stimulated by a high K^+^ medium containing 55 mM KCl for 1 min. Then, the cells were washed once in ice-cold PBS(–) and lysed with lysis buffer (25 mM Tris-HCl pH 7.5, 150 mM NaCl, 5 mM MgCl_2_, 1% NP-40, 1 mM DTT, 5% glycerol, and EDTA-free protease inhibitors). The lysates were centrifuged at 16,000 *g* for 15 min at 4°C, and the supernatant was collected. The protein concentrations were determined using a BCA Protein Assay Reagent Kit (Pierce). Activated Rac1 was detected by a pulldown assay using an EZ-detect Rac1 activation kit (Pierce), according to the manufacturer's protocol. Briefly, SwellGel-immobilized glutathione disks were placed into spin columns together with 20 μg of GST-human Pak1-PBD and cell lysates containing at least 1 mg of proteins. The reaction mixtures were incubated for 1 h at 4°C with gentle rocking. The resin was washed three times in the lysis buffer, followed by the addition of 50 μl of 2× SDS sample buffer and boiling for 5 min. The eluted fractions were collected and subjected to SDS–PAGE and Western blot analysis. To quantify the intensity of a Rac1-immunoreactive band, ECL reaction time and film exposure times were adjusted to maintain the signals within a linear dynamic range of detection.

### Statistical analysis

For comparisons between two groups and more than three groups, Student's *t-*test and one-way analysis of variance (followed by a *post hoc* Tukey-Kramer test) were used, respectively [Prism 4.0 and 9.0 (GraphPad Software) and JMP 5.1.2 (SAS Institute)]. The results were represented as the mean ± standard error of means (SEM).

## Results

### Palmitoylation of CaMKIγ facilitates association with lipid rafts

We reasoned that the addition of a palmitoyl moiety to prenylated wild-type CaMKIγ may facilitate the redistribution of CaMKIγ into specific membrane microdomains such as lipid rafts (Anderson and Jacobson, 2002). To substantiate this, we expressed CaMKIγ in COS7 cells with or without GODZ/DHHC3, a Golgi-enriched PAT that does not localize to lipid rafts. Under similar conditions, palmitate incorporation into CaMKIγ was increased by 4.3-fold (Takemoto-Kimura et al., 2007). We thus measured the amount of CaMKIγ in the Triton X-100-insoluble fractions, considered here as lipid rafts. We found that increased palmitoylation by GODZ co-expression significantly augmented the amount of CaMKIγ co-fractionated in the lipid raft fractions, identified using specific markers such as caveolin and flotillin (Figure 1A, top panels). In contrast, a palmitoylation-deficient 4CS (C417S, C419S, C420S, and C423S) mutant (Takemoto-Kimura et al., 2007) was completely absent from the raft fractions irrespective of the absence or presence of GODZ (Figure 1A, bottom panels).

We next investigated the effect of GODZ activity on CaMKIγ localization in cultured hippocampal neurons. The expression of GFP-CaMKIγ revealed a significant Golgi localization (identified using GM130 as a cis-Golgi marker) over a background of relatively diffuse cytoplasmic fluorescence (Figure 1B, –GODZ). Upon additional expression of HA-GODZ, however, an increased amount of GFP-CaMKIγ now appeared localized to the Golgi apparatus and in somatodendritic puncta near the plasma membranes, with a marked diminishment of any diffuse signals (Figure 1B, +GODZ). The Golgi localization of CaMKIγ induced by the expression of GODZ was inhibited by overnight treatment with 2-bromopalmitate (2-Br-Pal), a competitive inhibitor of PAT activity (Figure 1C). Furthermore, cholesterol depletion by methyl-beta-cyclodextrin (MeCD) also diminished the accumulation of CaMKIγ in the Golgi compartment (Figure 1D). These manipulations of palmitoylation and lipid raft states indicated that Golgi accumulation of CaMKIγ depends on both palmitoylation and intact cholesterol content on the Golgi membranes. Interestingly, throughout these manipulations, GODZ completely co-localized with a cis-Golgi marker, GM130, suggesting that GODZ localization to the Golgi is stable and may not depend on either palmitoylation or membrane cholesterol. Consistently, GODZ was not biochemically co-fractioned in the raft fractions either (Figure 1A).

Fluorescent localization of a prenylation-deficient mutant, C474S, which also lacks palmitoylation, shows a completely diffuse and cytosolic distribution devoid of any Golgi apparatus accumulation in accordance with our previous results (Figure 1E; Takemoto-Kimura et al., 2003, 2007). In contrast, the palmitoylation-deficient mutant 4CS, which is prenylated to a similar extent as the WT CaMKIγ (Takemoto-Kimura et al., 2007), showed undistinguishable localization in Golgi as compared with the wild type (Figure 1E). Together, we conclude that the prenylation of CaMKIγ is sufficient to enable its membrane insertion, permitting full access to the Golgi. Further association with a Golgi PAT, GODZ, may then facilitate CaMKIγ's subsequent palmitoylation that leads to its final association with lipid raft-like puncta.

### Raft association is necessary for the multimeric complex formation of CaMKIγ but does not affect Ca^2+^/CaM-dependent kinase activity

**Figure 2:**
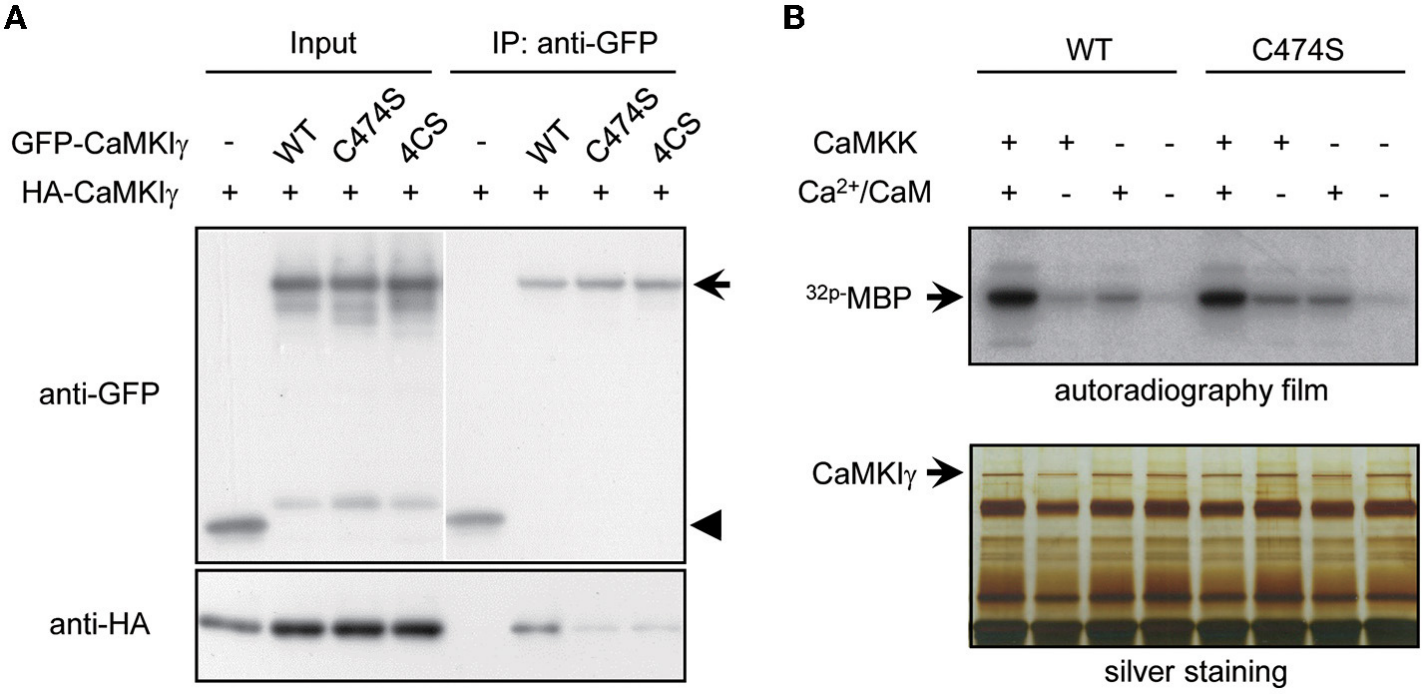
Palmitoylation is necessary for multimeric complex formation of CaMKIγ but not for membrane anchoring and kinase activity. **(A)** Palmitoylated CaMKIγ forms a multimeric complex in a heterologous cell system. Wild-type or mutant CaMKIγ were tagged with either HA or GFP, co-expressed in COS-7 cells, and co-immunoprecipitated using an anti-GFP antibody. The association of non-palmitoylated CaMKIγ mutants (C474S and 4CS) with wild-type CaMKIγ (HA- CaMKIγ) was substantially reduced. IP, immunoprecipitates. **(B)** Neither prenylation nor palmitoylation modulated the kinase activity of CaMKIγ. GFP-CaMKIγ-WT or C474S (a lipid modification-deficient mutant) was expressed in COS7 cells with or without the upstream kinase CaMKK and immunoprecipitated with an anti-GFP antibody, followed by an *in vitro* kinase assay using MBP as a substrate. When CaMKK was co-expressed, each kinase showed maximal kinase activity in the presence of Ca^2+^/CaM to a similar extent.

What is the biochemical significance of sequential lipid modifications—prenylation and palmitoylation—and consequential stepwise lipid membrane targeting? We tested whether these lipidation states facilitated the kinase's self-association or its kinase activity itself. Indeed, many signaling molecules localized to lipid rafts form multimers by lipidation (Zacharias et al., 2002; Huang and El-Husseini, 2005). Consistent with this, raft-enriched, HA-tagged CaMKIγ-WT was co-immunoprecipitated with GFP-CaMKIγ-WT. In contrast, non-palmitoylated CaMKIγ mutants, 4CS or non-lipidated C474S, were unable to assemble with GFP-CaMKIγ-WT (Figure 2A). Because the lipid-modified cysteine residues were located close to the regulatory domain of CaMKIγ, we next asked whether the lipidation states might affect its kinase activity. We evaluated the *in vitro* Ca^2+^/CaM-dependent kinase activity for WT and the lipidation-deficient mutant, C474S. In contrast to a previous finding that a kinase-deficient CaMKIγ had received a lesser amount of palmitoylation (Takemoto-Kimura et al., 2007), we here observed less difference with regard to CaMKK- and Ca^2+^-dependent kinase activity toward a substrate, MBP, between WT and the C474S mutant (Figure 2B). Together, these findings indicate that while the active conformation of CaMKIγ may be structurally beneficial to become a GODZ palmitoylation substrate on the Golgi membranes, the lipidation states may primarily modulate kinase self-association in the lipid rafts and not the kinase activity *per se*.

### The rat pheochromocytoma cell line PC12 cells possess multiple signal-activated neuritogenetic pathways and are deficient for CaMKIγ

**Figure 3:**
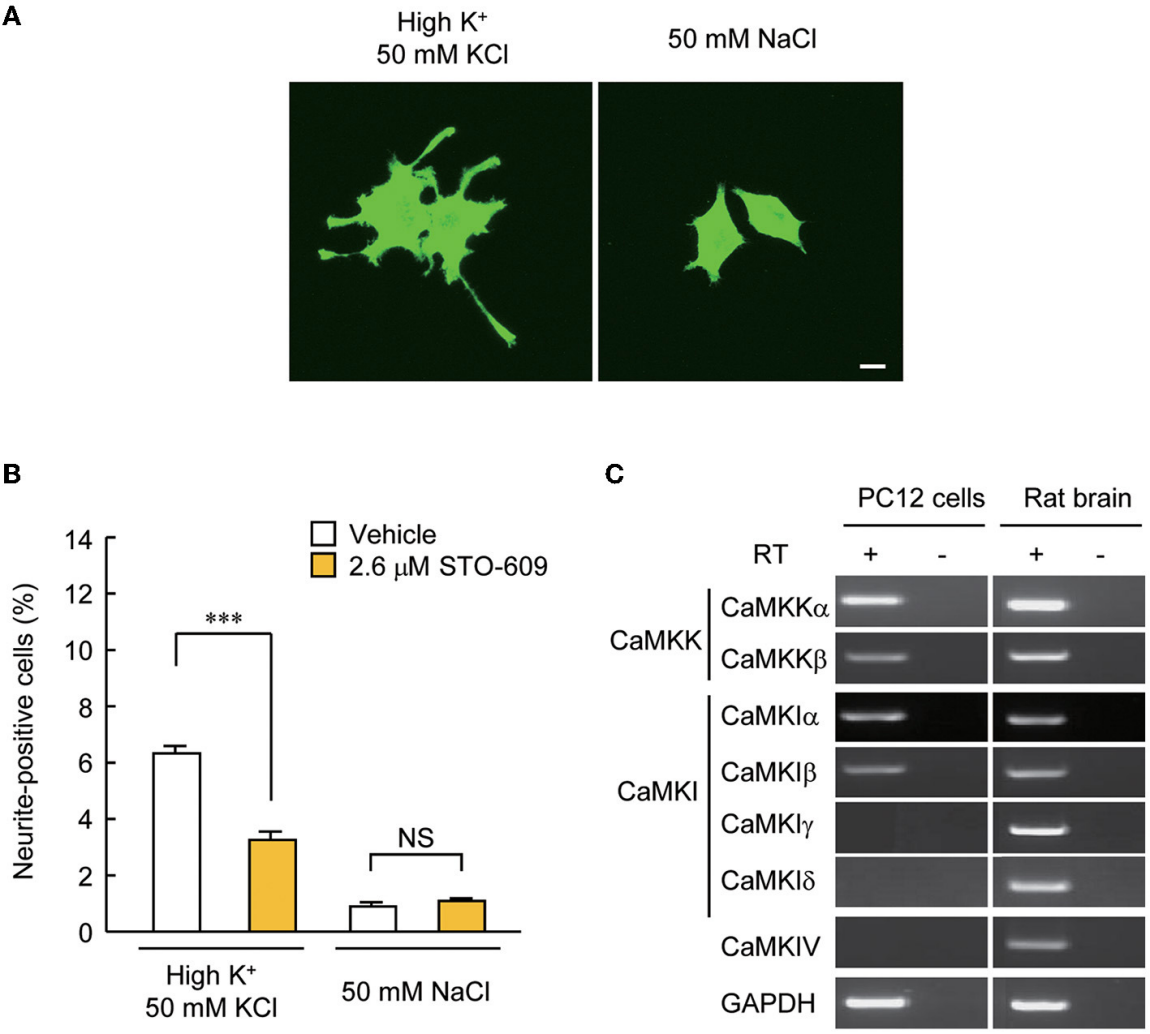
PC12 cells are a suitable model for testing the significance of CaMKIγ palmitoylation in recruiting lipid raft-restricted signaling underlying activity-dependent neuritogenesis. **(A)** Representative images of PC12 cells treated with neuritogenic stimuli: high-K^+^ solution, NGF, and forskolin. Neuritogenic activity was scored by counting the number of neurite-positive cells that harbored one or more neurites longer than their major cell body axis. The high-Na^+^ solution was a control experiment for high-K^+^ to adjust osmolality. Scale bar, 10 μm. **(B)** Blockade of an upstream kinase, CaMKK, by STO-609 inhibited high K^+^-induced neurite extension. *n* = 5, 5. ***p < 0.001. n.s., not significant. **(C)** CaMKK α/β, CaMKIα/β, but not CaMKIγ, were detected by RT-PCR in PC12 cells, while all kinases were expressed in the rat brain. Without the reverse transcriptase (RT) reaction, amplification was observed at the detection level, demonstrating no contamination with genomic DNA.

Our experiments so far pointed to the possibility that CaMKIγ is redistributed via palmitoylation to specific membrane signalosomes, such as lipid rafts (Anderson and Jacobson, 2002). We previously found that CaMKIγ palmitoylation is necessary for BDNF- and Ca^2+^-regulated dendritogenesis (Takemoto-Kimura et al., 2007). However, we then realized that further clean mechanistic dissection through functional rescue experiments was hampered in primary neurons because BNDF release is strongly stimulated downstream of BDNF-stimulated Ca^2+^ signaling in neurons. Thus, the direct examination of the significance of CaMKIγ palmitoylation on lipid raft signaling will be inevitably contaminated by TrkB-mediated lipid raft signaling triggered upon stimulation (Suzuki et al., 2004). To overcome this, we sought a tangible reconstitution mammalian cell model expressing a large number of neuronal PATs, including DHHC3/GODZ, a known PAT for CaMKIγ (Greaves and Chamberlain, 2011). Messenger RNAs for four neuronal PATs (DHHC3/GODZ, DHHC7, DHHC15, and DHHC17) were previously detected in PC12 cells (Greaves et al., 2008). Furthermore, PC12 cells fulfilled three advantageous conditions: first, numerous reports have previously shown activation of a lipid raft-associated signal complex in PC12 cells (e.g., Scott-Solomon and Kuruvilla, 2020). Second, depolarization by treatment with a 50 mM KCl solution known to induce Ca^2+^ influx via voltage-dependent Ca^2+^ channels (VDCC) (Banno et al., 2008) significantly increased the number of cells with neurites longer than their main cell body in PC12 cells (Figure 3A), enabling a rapid unbiased assay of activity-dependent neurite growth that was independent of any activity-dependent growth factor release. Finally, CaMKK was expressed in PC12 cells, and neurite extension triggered by depolarization was attenuated by inhibition of CaMKK with a specific inhibitor, STO-609 (Figure 3B). Using RT-PCR, we examined which CaMKK isoforms and which CaMKI or IV subtypes were expressed in PC12 cells. We found the expression of CaMKKα, CaMKKβ, CaMKIα, and CaMKIβ, but were unable to detect the amounts of CaMKIγ, δ, and CaMKIV mRNAs in PC12 cells (Figure 3C). These results suggested that Ca^2+^-CaMKK-dependent pathways may regulate activity-dependent neurite extension in PC12 cells through CaMKIγ-independent mechanisms. These experiments suggested that PC12 cells may offer a privileged condition to straightforwardly design and perform functional rescue experiments using distinct lapidated states of CaMKIγ.

### Palmitoylated CaMKIγ expression is sufficient to promote Ca^2+^-induced neuritogenic activity via a lipid raft-compartmentalized STEF-Rac1 pathway

**Figure 4:**
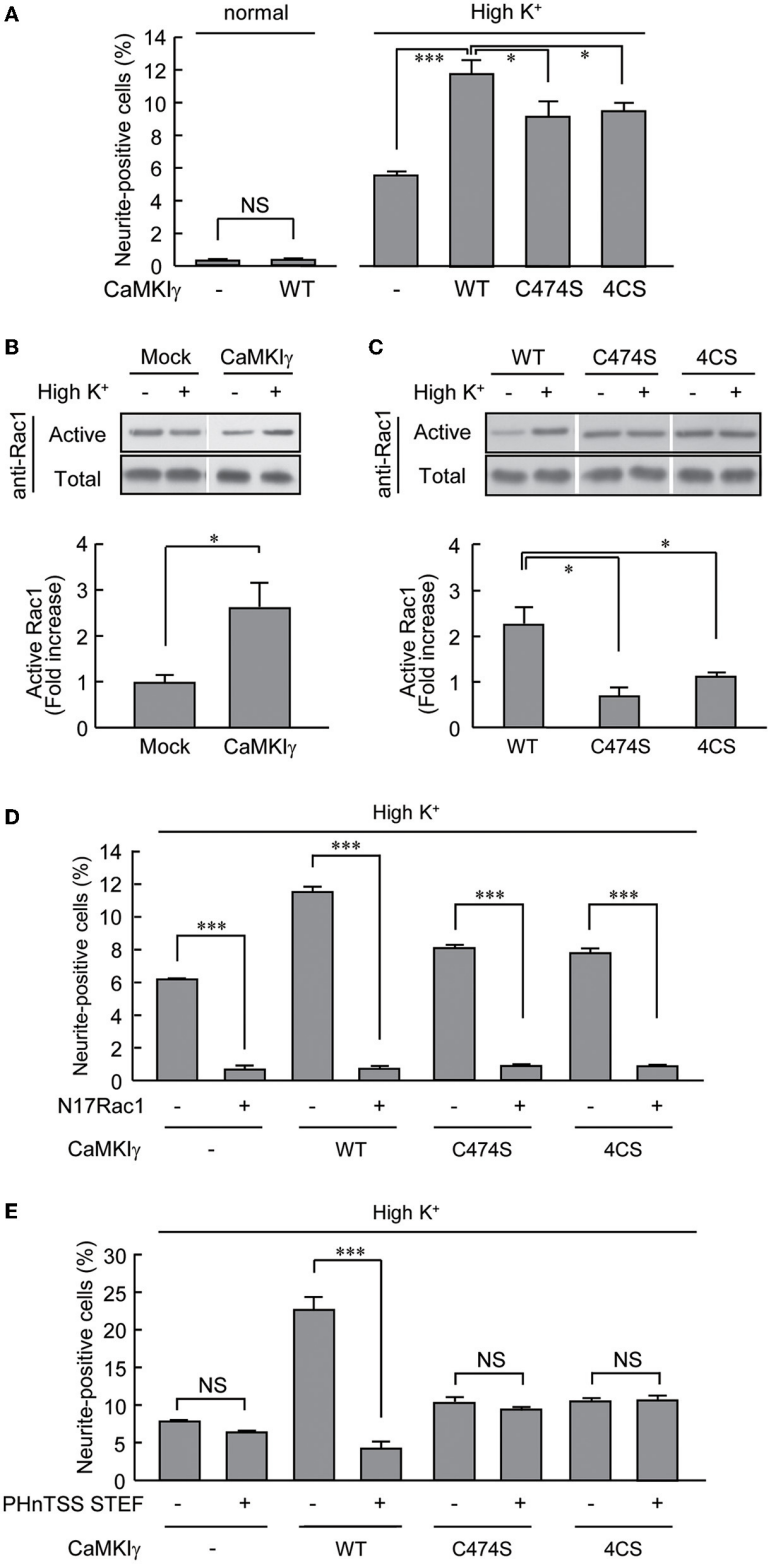
CaMKIγ expression is sufficient to promote neuritogenic activity in PC12 cells stimulated by prolonged high K^+^ stimuli. **(A)** A high K^+^ condition induces neurite extension but forced CaMKIγ expression under a high K^+^ condition further facilitates neuritogenic activity. PC12 cells were stimulated by high K^+^ (55 mM)-containing medium for 24 h, starting at 4 h after transfection. Lipid-modification mutants (C474S and 4CS, both lacking palmitoylation) of CaMKIγ showed significantly reduced neuritogenesis. The results represent the means ± SEM from four independent experiments. Normal medium: Mock, 0.28 ± 0.04; WT, 0.34 ± 0.02. High K^+^ medium: Mock, 5.49 ± 0.30; WT, 11.72 ± 0.74; C474S, 9.11 ± 0.87; 4CS, 9.42 ± 0.41. *n* = 5, 5, 5, 5, 5, 5. **(B)** High K^+^ stimulation led to significant Rac1 activation in CaMKIγ-expressing cells. Rac1 activation was measured by film densitometry (top) and indicated as the fold increase of GTP-bound active Rac1 (bottom). The total amount of Rac1 was not changed significantly (total, upper panel). All assays were performed 24 h after transfection. The results from three independent experiments are shown. Mock, 0.98 ± 0.18; CaMKIγ, 2.59 ± 0.54. **(C)** Expression of the lipid-modification mutants (C474S, 4CS) of CaMKIγ led to an impairment in high K^+^-induced Rac1 activation. The top panels show representative blots from Pak1-PBD pulldown assays, and the fold increase of GTP-bound active Rac1 was calculated as **(B)**. Results from three independent experiments are shown. WT, 2.23 ± 0.38; C474S, 0.69 ± 0.18; 4CS, 1.11 ± 0.10. *n* = 3, 3, 3. **(D, E)** A lipid microdomain-localized RacGEF-Rac1 pathway was essential for the palmitoylated CaMKIγ-dependent facilitation of neuritogenic activity. Expression of a dominant-negative Rac1 (N17Rac1) **(D)** and of a dominant negative fragment of STEF that disrupts proper membrane targeting of STEF (PHnTSS STEF) **(E)** strongly interfered with CaMKIγ-dependent neuritogenesis. However, the latter disrupted high K^+^-induced neuritogenesis only in CaMKIγ-expressing cells while sparing the cells lacking CaMKIγ. PC12 cells were stimulated as in **(A)** for the Rac1 experiment (N17Rac1). For the STEF experiment (PHnTSS STEF), the stimulations started at 24 h rather than 4 h, after transfection to achieve an optimal dominant-negative effect. The data are from three independent experiments. **(D)** Mock – N17Rac1, 6.13 ± 0.05; Mock + N17Rac1, 0.66 ± 0.18; CaMKIγ – N17Rac1, 11.46 ± 0.22; CaMKIγ + N17Rac1, 0.70 ± 0.12; CaMKIγ-C474S – N17Rac1, 8.17 ± 0.18; CaMKIγ-C474S + N17Rac1, 0.91 ± 0.02; CaMKIγ-4CS – N17Rac1, 7.82 ± 0.25; CaMKIγ-4CS + N17Rac1, 0.95 ± 0.01. *n* = 3, 3, 3, 3. **(E)** CaMKIγ – PHnTSS STEF, 7.84 ± 0.10; Mock + PHnTSS STEF, 6.40 ± 0.13; CaMKIγ – PHnTSS STEF, 22.83 ± 1.08; CaMKIγ + PHnTSS STEF, 4.19 ± 0.71; CaMKIγ-C474S – PHnTSS STEF, 10.33 ± 0.50; CaMKIγ-C474S + PHnTSS STEF, 9.48 ± 0.12; CaMKIγ-4CS – PHnTSS STEF, 10.36 ± 0.27; CaMKIγ-4CS + PHnTSS STEF, 10.93 ± 0.23. *n* = 3, 3, 3, 3. *p < 0.05; ***p < 0.001. n.s., not significant.

We took advantage of the PC12 cells, as these had no detectable levels of CaMKIγ expression. Overexpression of CaMKIγ-WT itself had no stimulatory effect on neurite extension (Figure 4A, left bars). While a 24-h exposure to high K^+^ solution significantly triggered neuritogenesis, the expression of CaMKIγ-WT in combination with a 24 h exposure to high K^+^ doubled the amount of neurite outgrowth compared to non-transfected cells, suggesting that Ca^2+^ signaling via heterologously expressed CaMKIγ can contribute to neurite formation, presumably downstream of endogenous CaMKK (Figure 4A, right bars). In contrast, this high K^+^-induced neurite outgrowth was significantly attenuated in cells expressing a palmitoylation-deficient mutant (4CS) or a prenylation-deficient mutant (C474S; Figure 4A), both of which also lacked palmitoylation. This confirmed that a sizable portion of neurite formation was elicited via reconstituted CaMKIγ signaling to require lipidation that might facilitate lipid raft recruitment and not via a simple augmentation of CaMKIγ kinase activity (Figure 2B).

**Figure 5:**
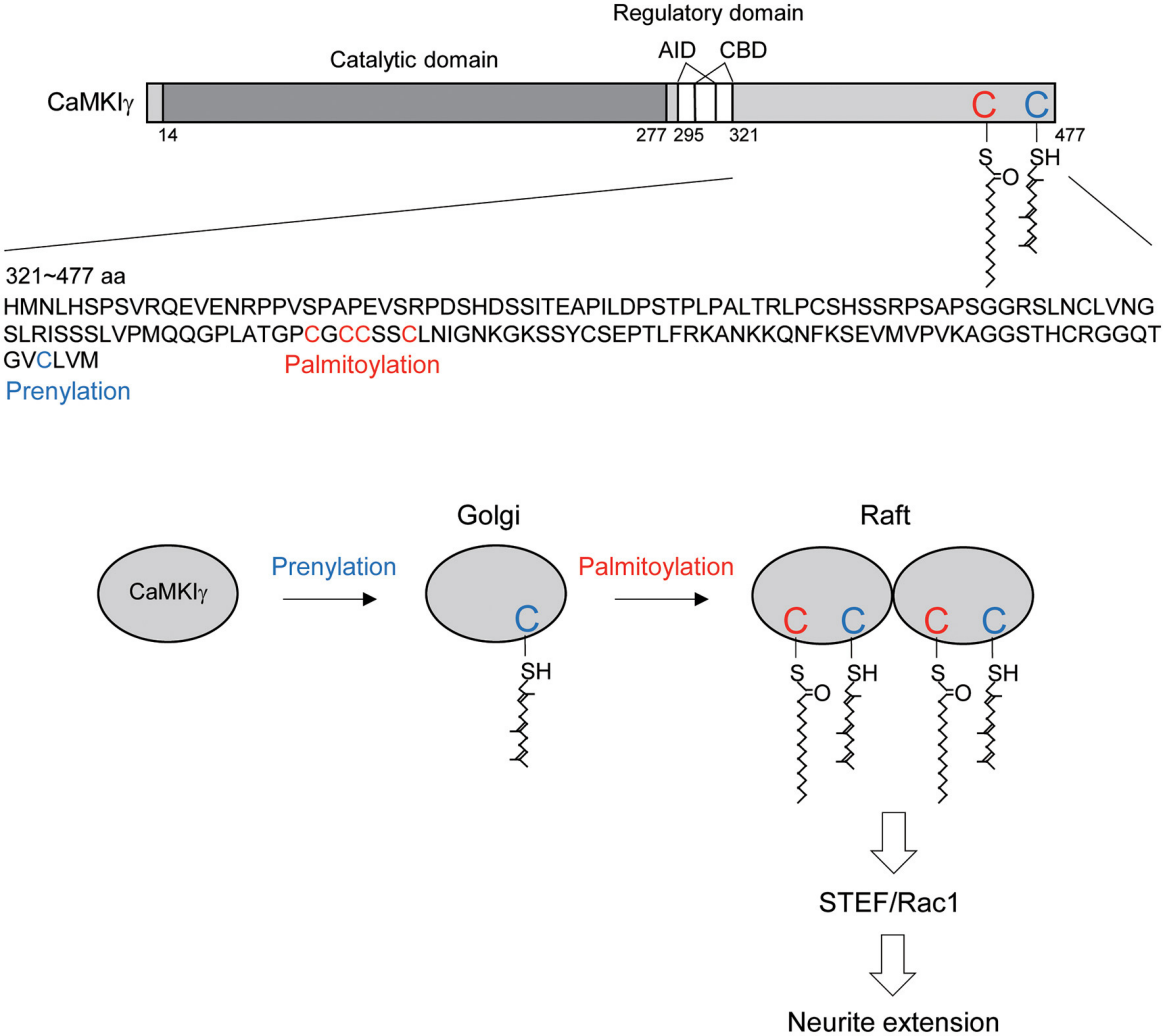
Schematic diagram illustrating the CaMKIγ C-terminal amino acid sequences and the proposed mechanistic model. **(Top)** CaMKIγ domain structure and amino acid sequences (321–477 aa). CaMKIγ undergoes prenylation on Cys-474 (blue letter) and then palmitoylation on Cys-417, Cys-419, Cys-420, and Cys-423 (red letters). AID, autoinhibitory domain, CBD, Ca^2+^/CaM binding domain. **(Bottom)** CaMKIγ localizes to the Golgi by undergoing prenylation. Subsequently, CaMKIγ is further palmitoylated, which then regulates multimer formation and raft localization. Lipid-modified CaMKIγ stimulates STEF and Rac1 activity, thus triggering increased neurite extension.

A robust increase in neuritogenesis in PC12 cells was likely to involve dynamic remodeling of the actin cytoskeleton, and we first tested whether Rac1 small GTPase activity was modulated by high K^+^ stimulation in the CaMKIγ-WT-expressing cells. Indeed, a significant rise in GTP-bound Rac1 was shown (Figure 4B). In sharp contrast, neither the C474S nor the 4CS mutants of CaMKIγ were able to stimulate Rac1 activity under high K^+^ conditions, suggesting that the CaMKIγ-mediated increase in Rac1 was mediated by palmitoylation and required its lipid raft recruitment (Figure 4C). Is Rac1 activation specifically downstream of palmitoylated CaMKIγ, or is it also required when the lipidation state of CaMKIγ is perturbed during high K^+^-triggered neuritogenesis? A dominant negative Rac1 mutant, N17Rac1, that inhibits RacGEF activity, completely abolished all forms of high K^+^-dependent neuritogenesis, suggesting that all forms of activity-dependent neuritogenesis, irrespective of the direct activation mechanism, required intact RacGEF activity (Figure 4D). A contrasting result was found, however, when we tested the overexpression of PHnTSS, a dominant interfering fragment of a lipid raft-associated RacGEF, STEF/Tiam2, which is known to be involved in neurite outgrowth (Hoshino et al., 1999; Matsuo et al., 2002; Kawauchi et al., 2003). While we found less effect of the blocking peptide overexpression on PC12 cells in the absence of CaMKIγ, a significant block of neurite formation was found in PC12 cells expressing palmitoylated wild-type forms of CaMKIγ. Furthermore, the STEF-blocking peptide overexpression showed no significant effect in lipidation-deficient mutant CaMKIγ-expressing cells (Figure 4E). Thus, a palmitoylated CaMKIγ may recruit lipid raft-compartmentalized RacGEF activity and functionally couple Ca^2+^/CaM to polarized actin cytoskeletal remodeling during neuritogenesis (Figure 5).

## Discussion

In this article, we showed that each lipid modification step confers distinct and additive properties on CaMKIγ-initial prenylation-induced membrane insertion permits access to the Golgi, and subsequent palmitoylation on the Golgi facilitates association with lipid rafts. Increased palmitoylation via GODZ augmented the accumulation of CaMKIγ on the Golgi in a cholesterol-dependent way. Interestingly, GODZ and CaMKIγ showed slightly different distributions on the Golgi: GODZ completely co-localized with GM130, while CaMKIγ showed more diffuse re-distribution within the Golgi area, indicating the following sequential membrane targeting model: (1) prenylation during CaMKIγ translation favors its membrane anchoring and its access to the Golgi apparatus; (2) transient association of CaMKIγ with GODZ on GM130-positive cis-Golgi membranes helps induce palmitoylation of CaMKIγ when the kinase activity is turned on by neuronal activity; (3) palmitoylated CaMKIγ then dissociates from GODZ and is in turn recruited into lipid raft microdomains and may form a signalosome complex on trans-Golgi rafts (Figure 5). This idea was also supported by the data from the biochemical fractionation of lipid rafts, where CaMKIγ but not GODZ is enriched in the raft fraction. It has been suggested that the raft platforms at the trans-Golgi network enable sphingolipids, sterols, and specific lipid raft proteins to assemble and be trafficked to their shared destination (Surma et al., 2012). By analogy to such raft-directed protein trafficking mechanisms, we propose that palmitoylated CaMKIγ may be recruited into the lipid microdomain within the Golgi, thus facilitating the formation of a signaling platform for trafficking to the dendritic microdomain (Takemoto-Kimura et al., 2007).

Our results suggest that palmitoylated CaMKIγ can localize in lipid rafts and form complexes with a lipid raft-compartmentalized RacGEF to phosphorylate this RacGEF or specifically recruit RacGEF into lipid rafts, and thus trigger a polarized Rac1 activity that elicits a neuritogenetic response involving polarized membrane trafficking and cytoskeletal remodeling. CaMKIγ may share multiple lipid modification mechanisms with Ras and RhoGTPases (e.g., H-Ras, N-ras, and Rac1): all of these undergo prenylation on the C-terminal Caax motif (farnesylation for Ras and geranylgeranylation for RhoGTPases), and subsequent adjacent palmitoylation sites have been shown. H-Ras is known to shuttle between the Golgi and the plasma membrane microdomains via an active prenylation-palmitoylation-depalmitoylation cycle, which is essential for its function in the plasma membranes (Eisenberg et al., 2013). Recently, Rac1 palmitoylation was also shown to be important for actin cytoskeleton remodeling by controlling plasma membrane organization (Navarro-Lerida et al., 2012). Thus, palmitoylation-dependent Golgi-plasma membrane trafficking may underlie in part the function of CaMKIγ at the microdomains in the peripheral plasma membrane.

On the other hand, palmitoylation of the transmembrane domain 2 of AMPA receptors was associated with a reduction of their surface expression levels (Hayashi et al., 2005). Therefore, it is also possible that palmitoylated CaMKIγ is preferentially recruited within the microdomain in the Golgi, where they may act on the localized STEF-Rac1 pathway to regulate neurite extension. As Golgi outposts were shown to regulate dendritic development (Horton et al., 2005; Ori-McKenney et al., 2012), this possibility aligns well with the dendritic phenotype of CaMKIγ knockdown/knockout neurons (Takemoto-Kimura et al., 2007). Further studies are clearly needed to further substantiate the contribution of each of these possible hypotheses.

Neuronal morphogenesis, which is regulated by neuronal Ca^2+^ (Rosenberg and Spitzer, 2011), seems to rely, at least in part, on the signaling cascade at the lipid rafts (Hering et al., 2003; Takemoto-Kimura et al., 2007), but the molecular mechanisms at the lipid rafts that link Ca^2+^ and downstream morphological changes are still largely unknown. Our result identified a sequential prenylation-palmitoylation-dependent molecular mechanism that specifies CaMKIγ recruitment to lipid microdomains and facilitates physical association and functional coupling with a compartmentalized Rac1 pathway to mediate Ca^2+^-dependent neuritogenesis. Our results confirm prior results and further suggest that elucidating the lipidation code of key signaling molecules may shed light on hitherto elusive membrane targeting and recruitment mechanisms that may critically operate in neurons during axodendritic polarization and help specify the outcomes of Ca^2+^-dependent neuronal morphogenesis and circuitogenesis.

## Data availability statement

The raw data supporting the conclusions of this article will be made available by the authors, without undue reservation.

## Ethics statement

The animal study was reviewed and approved by Animal Experimentation Committee of the University of Tokyo Graduate School of Medicine and Toho University Animal Care and User Committee.

## Author contributions

NA-I and ST-K conducted the biochemical and morphological analyses. YK and MO helped with the biochemical analysis. ST-K and HB supervised the projects. NA-I, ST-K, and HB wrote the manuscript. All authors read and approved the final manuscript.
